# Predictors of initiation and persistence of unhealthy weight control behaviours in adolescents

**DOI:** 10.1186/1479-5868-6-72

**Published:** 2009-10-29

**Authors:** Jennifer A Linde, Melanie M Wall, Jess Haines, Dianne Neumark-Sztainer

**Affiliations:** 1 Division of Epidemiology and Community Health, School of Public Health, University of Minnesota, Minneapolis, MN, USA; 2 Division of Biostatistics, School of Public Health, University of Minnesota, Minneapolis, MN, USA; 3 Department of Ambulatory Care and Prevention, Harvard Medical School/Harvard Pilgrim Health Care, Boston, MA, USA

## Abstract

**Background:**

Unhealthy weight control behaviours (UWCB) among adolescents have significant health and weight consequences. The current longitudinal study aimed to identify personal and socio-environmental predictors of initiation or persistence of adolescent UWCB, in order to inform development of programs aimed at both preventing and stopping UWCB.

**Methods:**

A diverse sample included 1106 boys and 1362 girls from 31 middle schools and high schools in the United States who were enrolled in Project EAT (Eating Among Teens). Project EAT explored personal, behavioural, and socio-environmental factors associated with dietary intake and body weight in adolescence. Participants completed questionnaires to assess demographics, UWCB (including several methods of food restriction, purging by vomiting or medications, smoking to control weight, or food substitutions) and personal and socio-environmental variables at two time points, five years apart, between 1998 and 2004. Logistic regression models examined personal and socio-environmental predictors of initiation and persistence of UWCB among Project EAT participants.

**Results:**

Results indicate that 15.5% of boys and 19.7% of girls initiated UWCB by Time 2, and 15.9% of boys and 43.3% of girls persisted with these behaviours from Time 1 to Time 2. After controlling for race/ethnicity and weight status changes between assessments, logistic regression models indicated that similar factors and patterns of factors were associated significantly with initiation and persistence of UWCB. For both boys and girls, personal factors had more predictive value than socio-environmental factors (Initiation models: for boys: *R*^2 ^= 0.35 for personal vs. 0.27 for socio-environmental factors; for girls, *R*^2 ^= 0.46 for personal vs. 0.26 for socio-environmental factors. Persistence models: for boys: *R*^2 ^= 0.53 for personal vs. 0.33 for socio-environmental factors; for girls, *R*^2 ^= 0.41 for personal vs. 0.19 for socio-environmental factors). The weight concerns model was the strongest predictor among all individual models [Initiation odds ratios (ORs) and 95% confidence interval (CI): 4.84 (3.32-7.01) for boys and 5.09 (3.55-7.30) for girls; persistence OR (CI): 4.55 (2.86-7.14) for boys and 3.45 (2.50-4.76) for girls].

**Conclusion:**

In general, predictors of initiation and persistence of UWCB were similar, suggesting that universal and selective prevention programs can target similar risk factors.

## Background

A high percentage of adolescents engage in unhealthy behaviours to control weight. These unhealthy behaviours include purging (by vomiting or laxative use), use of diet pills, skipping meals, and smoking to control weight [1-5]. National surveys of adolescents in the United States indicate self-reported lifetime engagement in specific unhealthy weight control behaviours (UWCB) ranging from 4-18% of boys and 10-49% of girls [2,3,5]; in other national U.S. surveys, 30-day prevalence rates for at least one UWCB range from 10-11% of boys and 22-26% of girls [1,4]. Concerns about use of these unhealthy behaviours are warranted. Such behaviours have serious negative consequences, including weight gain over time and poorer dietary intake [6-10] and may place adolescents at risk for the development of clinically significant eating disorders [8,11-13].

Personal and socio-environmental correlates of UWCB have been observed in multiple studies. Among personal factors, associations have been observed for self-esteem and depression [14,15] and body dissatisfaction or weight concerns [16-19]. Socio-environmental factors associated with UWCB include media exposure [20,21], parental weight concerns [14,21-23], poor family communication [22], and peer attitudes [14,15,20,23]. Comparable associations have been reported in the United States [14,15,20,21], United Kingdom [22] and Australia [19,23].

An earlier cross-sectional analysis of the data to be used in the current longitudinal analyses found that weight and body concerns, family and peer weight norms, and family connectedness (a composite variable that takes family communication into account) were moderate to strong correlates of UWCB [24]. However, differences in associations of these factors with initiation or persistence of UWCB over time were not examined in the earlier cross-sectional study [24]. In addition, questions of how to approach intervention development to prevent or halt UWCB use arise. Should we consider only weight and body concerns, which were the strongest correlates of baseline levels of UWCB in this sample [24], or might other associated factors be appropriate targets for intervention? Additionally, are there divergent determinants of initiation or persistence of UWCB that might inform intervention decisions based on UWCB status? That is, within prevention efforts, can we include adolescents who are not using UWCB with adolescents already engaging in these behaviours? The present study is an examination of predictors of initiation or persistence of UWCB over five years. This study is intended to elucidate differential patterns of UWCB predictors to inform future intervention development.

## Methods

### Study Design and Participants

Data were drawn from Project EAT (Eating Among Teens), which studied personal, behavioural, and socio-environmental factors associated with dietary intake and weight-related outcomes in adolescence [25,26]. Project EAT-I assessed an ethnically diverse cohort of 4746 adolescents from 31 middle schools and high schools in the Midwestern United States, starting in 1998. Project EAT-II attempted to survey 3633 Project EAT-I participants for whom contact information was provided, and was successful in obtaining mailed data from 2516 adolescents (53.0% of original Project EAT-I cohort, 68.4% of those contacted for Project EAT-II) after five years.

The Institutional Review Board at the investigators' home university approved study protocols for both phases of Project EAT. Consent involved separate protocols for participants under 18 years of age and for young adult participants in Project EAT-II who had progressed to 18 years of age or beyond. Parents of those under 18 received a letter of consent before surveys were mailed to adolescents. If the letter was not returned with a refusal of participation by a parent, a survey was mailed to the adolescent along with a letter of assent to be returned with the completed survey; the adolescent was given the option to return the letter of assent without the survey as a means of declining participation. For those ages 18 years or older, a survey was mailed directly to the potential participant and consent was indicated by return of the survey to study staff.

### Measures

#### UWCB outcomes

UWCB were assessed by self-report on Time 1 (Project EAT-I) and Time 2 (Project EAT-II) surveys, with five years between surveys. These behaviours included fasting, eating very little food, taking diet pills, induced vomiting, laxative use, diuretic use, use of food substitutes (e.g., powdered meal replacement products), skipping meals, and smoking more cigarettes than usual as a means of weight control. Adolescents were asked to indicate whether they had engaged in each behaviour to lose weight or prevent weight gain during the past year (yes/no response format). For the purpose of this study, adolescents were classified as engaging in UWCB if they responded "yes" to at least one behaviour. Prevalence of UWCB (aggregated and by individual behaviours) at Time 1 in this sample and changes in UWCB engagement over time are reported elsewhere [8,27].

#### Personal and socio-environmental factors

Personal and socio-environmental factors were defined for Project EAT surveys using concepts from social cognitive theory, which argues that personal characteristics interact with the physical and/or social environment to determine behaviour [28]. Constructs were assessed as follows:

### Personal Factors

#### Self-esteem

Assessed using six items from the Rosenberg Self-Esteem scale [29]. Cronbach's α = .79 in this sample.

#### Depressed mood

Assessed using six items from the Kandel and Davies [30] depressive mood scale. Cronbach's α = .82 in this sample.

#### Weight concerns

Assessed by two items: "I think a lot about being thinner," "I am worried about gaining weight" (4-point Likert scale, strongly disagree to strongly agree). Items were developed for Project EAT [25,26]. Cronbach's α = .87 in this sample.

#### Body dissatisfaction

Assessed satisfaction with 10 body parts on 5-point Likert scales (very dissatisfied to very satisfied), using items based on the Body Shape Satisfaction scale [31] and the Body Cathexis Scale [32]. Cronbach's α = .92 in this sample.

#### Weight/shape importance

"During the past six months, how important has your weight or shape been in how you feel about yourself? (not very important, played a part, among the main things, most important things). This item was modified from the Questionnaire on Eating and Weight Patterns- Revised [33].

#### Benefits of healthy eating

"The types of food I eat affect: my health, how I look, my weight, how well I do in sports, how well I do in school" (5-point Likert scale, strongly disagree to strongly agree for each item). Items were developed for the TEENS project [34]. Cronbach's α = .83 in this sample.

#### Health concerns

"How much do you care about: eating healthy foods, being healthy?" (not at all, a little, somewhat, very much for each of the two items) and "How strongly do you agree with the following statements? Teenagers don't need to be concerned about their eating habits; At this point in my life, I am not very concerned about my health; Teenagers don't need to worry about their health." (4-point Likert scale, strongly disagree to strongly agree for each of the three items). These items were developed for Project EAT [25,26]. Cronbach's α = .70 in this sample.

### Socio-environmental Factors

#### Parents' concerns about weight

"My mother (or father) diets to lose weight or keep from gaining weight," and "My mother (father) encourages me to diet to control my weight" (not at all, a little, somewhat, very much). The items were developed for Project EAT [25,26]. Cronbach's α = .77 in this sample.

#### Peer dieting

"Many of my friends diet to lose weight or keep from gaining weight" (not at all, a little, somewhat, very much, don't know). This item was developed for Project EAT [25,26].

#### Teasing frequency

"How often do any of the following things happen? You are teased about your weight" (never, less than once a year, a few times a year, a few times a month, at least once a week). This item was developed for Project EAT [25,26].

Frequency of reading dieting or weight loss articles (weight loss article reading): "How often do you read magazine articles in which dieting or weight loss are discussed?" (never, hardly ever, sometimes, often). The item was developed for Project EAT [25,26].

#### Family connectedness

"How much do you feel you can talk to your mother (father) about your problems? How much do you feel your mother (father) cares about you?" (not at all, a little, somewhat, very much); items were adapted from the Voice of Connecticut Youth survey [35]. Cronbach's α = .69 in this sample.

#### Covariates

Study cohort was identified by age and grade level in school at study entry [older = high school students (grades 9-12), mean age 15.8 ± 0.8 years, range 14-18 years vs. younger = middle school students (grades 6-8), mean age 12.8 ± 0.8 years, range 11-14 years]. Race/ethnicity (Black/African American, Hispanic/Latino, Asian American, American Indian/Native American, Mixed or other, White) was self-reported. Self-reported height and weight were used to calculate body mass index (BMI; kg/m^2^). Weight status (never overweight; overweight at Time 1 only; overweight at Time 2 only; overweight at both Time 1 and Time 2) was defined using data from the first United States National Health and Nutrition Examination Survey (NHANES I) [36,37]; the threshold for overweight was defined as BMI at or above the 85th percentile for age and gender. This classification system has been used in all longitudinal examinations of Project EAT data [e.g., [6,8,27,38]] because it accounts for both adolescent and young adult values by providing 85th percentile curves for BMI from childhood through adulthood, and this sample ranged from 11-23 years of age during the study. In this sample, age and gender-normed BMI percentiles [36,37] indicate that 29.6% of boys and 31.8% of girls in the younger cohort were overweight at Time 1, and 24.3% of boys and 22.5% of girls in the older cohort were overweight at Time 1. At Time 2, 26.1% of boys and 32.7% of girls in the younger cohort were overweight, and 24.1% of boys and 25.0% of girls in the older cohort were overweight. Mean BMI values were 22.4 kg/m^2 ^for girls and 22.5 kg/m^2 ^for boys at Time 1, and 23.9 kg/m^2 ^for girls and 24.6 kg/m^2 ^for boys at Time 2.

### Data Analysis

Analyses were conducted using SAS software (version 9.1, 2003, SAS Inc., Cary, NC). Data were split into two groups determined by UWCB engagement patterns between Time 1 and Time 2. One group of analyses compared adolescents with no UWCB at Time 1, but UWCB at Time 2, with adolescents who reported no UWCB on either survey (initiation vs. never-engaging). The second group of analyses compared adolescents who reported engagement in UWCB on both surveys with those who reported UWCB at Time 1 only (persistence vs. cessation). For each group, logistic regression models were used to examine the association between each baseline predictor (Time 1) and change in the predictor (Time 2 - Time 1) on UWCB use at Time 2. Based on earlier conceptual modelling of UWCB correlates in this sample [24], predictors were classified as personal or socio-environmental (as described in *Measures*). Each personal or socio-environmental predictor (Time 1 value and change value) was entered into a separate model to examine its association with UWCB status at Time 2, adjusting for cohort, race/ethnicity, and weight status from Time 1 to Time 2. One set of these 12 models examined the impact of each predictor on initiation versus never engaging in UWCB between Time 1 and Time 2, and the second set of models examined the impact of each predictor on persistence versus cessation of UWCB by Time 2. We investigated the potential for clustering due to sampling within 31 schools and found no significant intraclass correlations; thus, we did not analyze the data using hierarchical models.

Odds ratios were considered statistically significant (*p *< .05) when 1.0 was not included in the 95% confidence interval. Max-rescaled *R*^2^, which is a function of the likelihood ratio test in logistic regression that measures the explanatory (predictive) value of a model [39], was calculated for each individual predictor model. Max-rescaled *R*^2 ^was also calculated from logistic regression models that examined all personal factors and adjustment variables entered together, all socio-environmental factors and adjustment variables entered together, all predictors (personal, socio-environmental, and adjustment variables) entered together, and adjustment variables only. As this version of *R*^2 ^is a function of the likelihood ratio test, the maximum possible value of *R*^2  ^in this case is not 1.0 as it is for linear regression, and max-rescaled *R*^2 ^divides by this maximum value so that the *R*^2 ^maximum is 1.0 [39].

Analyses, including 1106 boys and 1362 girls, were stratified by gender. To account for differential response rates across demographic characteristics in the longitudinal sample, the data were weighted using the response propensity method [40], in which the inverse of the estimated probability that an individual responded at Time 2 is used as the weight. The weighting method results in estimates that are representative of the demographic make-up of the Project EAT-I sample. Weighted ethnic/racial and SES proportion are: 48.5% White, 19.0% African-American, 19.2% Asian, 5.8% Hispanic, 3.5% Native American and 3.9% mixed or other race. Thirty-seven percent of adolescents were of low or low-middle socio-economic status. As previous work from Project EAT has shown that the personal and socio-environmental factors examined here are associated with overweight [6], that UWCB confer risk of increased BMI over time [8] and that UWCB and overweight status share comparable risk and protective factors [9], we treat weight status over time as a covariate to focus on the predictive value of personal and socio-environmental factors in determining UWCB status while controlling for the known association of weight with UWCB [8].

## Results

### Overview of Engagement in Unhealthy Weight Control Behaviours

At Time 1, 28.5% of boys (*n *= 315) and 56.1% of girls (*n *= 764) engaged in UWCB. By Time 2, an additional 15.5% of boys (*n *= 171) and 19.7% of girls (*n *= 268) had begun to engage in UWCB (initiation), and 15.9% of boys (*n *= 176) and 43.3% of girls (*n *= 589) had maintained their Time 1 engagement in UWCB (persistence).

### Predictors of Initiation versus Never Engaging in Unhealthy Weight Control Behaviours

Table 1 presents odds ratios (ORs) from 12 individual predictor models for associations of personal and socio-environmental factors with initiating UWCB between Time 1 and Time 2. Examination of the models indicates that boys and girls who initiated UWCB between Time 1 and Time 2, as compared to never-engagers, reported relatively greater personal concerns and socio-environmental pressures at Time 1. Among the personal factors, depressed mood (boys' OR = 1.57, girls' OR = 1.52), weight concerns (boys' OR = 4.84, girls' OR = 5.09), body dissatisfaction (boys' OR = 1.05, girls' OR = 1.07), importance of weight and shape (boys' OR = 1.71, girls' OR = 2.23), and lower self-esteem (boys' OR = 0.58, girls' OR = 0.71) at Time 1 all were associated with greater likelihood of UWCB initiation by Time 2.

**Table 1:** Associations of personal and socio-environmental factors with initiation of unhealthy weight control behaviours.

	**Initiation (vs. Never-engaging)**					
	
	**Boys**			**Girls**		
**Predictor**	**OR**	**95% CI**	***R*^2^**	**OR**	**95% CI**	***R*^2^**
Personal Factors			0.35			0.46
Self-esteem	**0.58**	0.44-0.75	0.16	**0.71**	0.55-0.91	0.15
Change in self-esteem	**0.89**	0.83-0.95		**0.86**	0.80-0.92	
Depressed mood	**1.57**	1.22-2.03	0.15	**1.52**	1.19-1.94	0.16
Change in depressed mood	**1.14**	1.06-1.23		**1.18**	1.10-1.26	
Weight concerns	**4.84**	3.32-7.04	0.31	**5.09**	3.55-7.30	0.43
Change in weight concerns	**3.64**	2.70-4.91		**5.87**	4.21-8.17	
Body dissatisfaction	**1.05**	1.01-1.08	0.16	**1.07**	1.04-1.11	0.20
Change in dissatisfaction	**1.06**	1.03-1.09		**1.08**	1.06-1.11	
Weight and shape importance	**1.71**	1.29-2.25	0.17	**2.23**	1.59-2.84	0.20
Change in importance	**1.85**	1.45-2.37		**2.30**	1.78-2.97	
Benefits: healthy eating	1.16	0.91-1.49	0.14	**1.51**	1.18-1.93	0.13
Change in benefits	**1.50**	1.12-2.01		**1.81**	1.33-2.46	
Health concerns	**1.30**	1.03-1.65	0.13	0.99	0.79-1.25	0.10
Change in health concerns	**1.10**	1.01-1.19		1.03	0.94-1.12	

Socio-Environmental Factors			0.27			0.26
Parental concern about weight	**1.64**	1.24-2.16	0.16	**1.42**	1.07-1.89	0.11
Change in parental concern	**1.09**	1.01-1.17		1.03	0.95-1.12	
Peer dieting	**1.98**	1.45-2.70	0.20	**1.57**	1.20-2.04	0.13
Change in peer dieting	**1.37**	1.10-1.70		**1.38**	1.14-1.68	
Teasing frequency	**1.40**	1.07-1.85	0.13	1.22	0.92-1.61	0.10
Change in teasing frequency	1.14	0.93-1.39		1.21	0.92-1.61	
Weight loss article reading	**1.68**	1.23-2.27	0.16	**1.92**	1.49-2.48	0.21
Change in reading	**1.71**	1.36-2.16		**2.07**	1.67-2.58	
Family connectedness	0.90	0.72-1.13	0.13	**0.70**	0.56-0.88	0.12
Change in connectedness	0.97	0.91-1.03		**0.93**	0.87-0.98	

Combined Models						
All predictors (personal and socio-environmental)			0.45			0.51
Covariates only (cohort, race, weight status)			0.12			0.10

Note. *N *= 791 for boys, 598 for girls. All models are adjusted for cohort, race/ethnicity, and weight status from Time 1 to Time 2. *R*^2 ^= percent variance explained by separate and combined predictor models. Each of 12 single predictor models includes predictor, change in predictor, and covariates. Combined models include all personal, all socio-environmental, or all predictors as well as change in predictor and covariates. Statistically significant odds ratios (*p *< .05) are shown in **bold **type.

Among socio-environmental factors, parental concern about weight (boys' OR = 1.64, girls' OR = 1.42), peer dieting (boys' OR = 1.98, girls' OR = 1.57), and weight loss article reading (boys' OR = 1.68, girls' OR = 1.92) at Time 1 all were associated with greater likelihood of UWCB initiation. For boys, higher rates of health concerns (personal, OR = 1.30) and weight teasing (socio-environmental; OR = 1.40) also were associated with initiating UWCB; for girls, having a stronger perception of the benefits of healthy eating (personal; OR = 1.51) and less family connectedness (socio-environmental; OR = 0.70) also were associated with initiating UWCB. Among the 12 individual predictors, higher levels of weight concerns at Time 1 were associated with the greatest odds of initiating UWCB.

With several exceptions (health concerns, parental concern about weight, weight teasing, and family connectedness), changes in the individual predictor variables from Time 1 to Time 2 also were associated significantly with initiation of UWCB by Time 2 for boys and girls; odds ratios were of comparable magnitude to those for the individual predictor values at Time 1. For boys only, increased health concerns (personal, OR = 1.10) and increased parental concern about weight (socio-environmental, OR = 1.09) were associated with UWCB initiation. For girls only, increased perception of the benefits of healthy eating (personal, OR = 1.51) and decreased family connectedness (socio-environmental, OR = 0.93) was associated with UWCB initiation. As with the individual Time 1 predictor values, increases in weight concerns from Time 1 to Time 2 had the strongest association with UWCB initiation by Time 2 (boys' OR = 3.64, girls' OR = 5.87).

Examination of max-rescaled *R*^2 ^values in Table 1 indicates high explanatory value of combined models that included either all personal or all socio-environmental factors (*R*^2 ^= 0.35 and 0.46 for personal factors and *R*^2 ^= 0.27 and 0.26 for socio-environmental factors for boys and girls, respectively); the explanatory value of the model with all predictors and adjustment variables entered was higher still (*R*^2 ^= 0.45 for boys, 0.51 for girls; see Combined Models section at bottom of Table 1). The individual model with the greatest predictive value in explaining UWCB initiation was the weight concerns model (*R*^2 ^= 0.31 for boys, 0.43 for girls). In individual models, the remaining personal predictors demonstrated half as much predictive power or less. For the individual socio-environmental factor models, peer dieting (*R*^2 ^= 0.20 for boys, 0.13 for girls) and weight loss article reading (*R*^2 ^= 0.16 for boys, 0.21 for girls) had the greatest predictive value in explaining UWCB initiation.

### Predictors of Persistence versus Cessation of Unhealthy Weight Control Behaviours

Table 2 presents odds ratios for associations of personal and socio-environmental factors with UWCB persistence from Time 1 to Time 2. Examination of the 12 individual predictor models indicates that boys and girls who persisted with UWCB, as compared to those who ceased UWCB, reported a pattern of concerns similar to that of UWCB initiators. For personal factors, those who persisted with UWCB reported a relatively greater degree of depressed mood (boys' OR = 1.85, girls' OR = 1.72), weight concerns (boys' OR = 4.55, girls' OR = 3.45), body dissatisfaction (boys' OR = 1.10, girls' OR = 1.08), preoccupation with weight and shape (boys' OR = 2.50, girls' OR = 2.44), and perceived benefits of healthy eating (boys' OR = 1.64, girls' OR = 1.39); for girls only, lower self-esteem was associated with persistence as well (OR = 0.54).

**Table 2:** Associations of personal and socio-environmental factors with persistence in unhealthy weight control behaviours.

	**Persistence (vs. Cessation)**					
	
	**Boys**			**Girls**		
**Predictor**	**OR**	**95% CI**	***R*^2^**	**OR**	**95% CI**	***R*^2^**
Personal Factors			0.53			0.41
Self-esteem	0.73	0.53-1.00	0.23	**0.54**	0.42-0.69	0.16
Change in self-esteem	0.94	0.87-1.01		**0.84**	0.79-0.89	
Depressed mood	**1.85**	1.33-2.50	0.28	**1.72**	1.37-2.22	0.16
Change in depressed mood	**1.18**	1.08-1.28		**1.25**	1.16-1.33	
Weight concerns	**4.55**	2.86-7.14	0.42	**3.45**	2.50-4.76	0.26
Change in weight concerns	**4.55**	2.86-7.14		**3.03**	2.38-4.00	
Body dissatisfaction	**1.10**	1.05-1.15	0.30	**1.08**	1.05-1.11	0.19
Change in dissatisfaction	**1.06**	1.03-1.10		**1.09**	1.06-1.12	
Weight and shape importance	**2.50**	1.79-3.57	0.31	**2.44**	1.89-3.23	0.21
Change in importance	**1.67**	1.27-2.22		**2.38**	1.89-3.03	
Benefits: healthy eating	**1.64**	1.19-2.27	0.26	**1.39**	1.10-1.79	0.13
Change in benefits	**1.92**	1.32-2.86		**2.27**	1.70-3.03	
Health concerns	1.14	0.86-1.49	0.21	1.01	0.82-1.25	0.08
Change in health concerns	1.06	0.95-1.18		1.04	0.96-1.12	

Socio-Environmental Factors			0.33			0.19
Parental concern about weight	1.14	0.86-1.49	0.19	**1.43**	1.12-1.82	0.10
Change in parental concern	1.04	0.96-1.12		**1.11**	1.04-1.21	
Peer dieting	**1.47**	1.04-2.08	0.25	**1.56**	1.22-2.00	0.10
Change in peer dieting	1.32	0.99-1.75		**1.47**	1.22-1.79	
Teasing frequency	**1.61**	1.18-2.22	0.25	**1.35**	1.05-1.64	0.10
Change in teasing frequency	**1.35**	1.10-1.67		**1.37**	1.14-1.64	
Weight loss article reading	**1.96**	1.33-2.94	0.27	**1.79**	1.41-2.27	0.12
Change in reading	1.30	0.96-1.79		**1.33**	1.10-1.61	
Family connectedness	0.81	0.61-1.06	0.21	0.83	0.66-1.03	0.08
Change in connectedness	0.94	0.89-1.01		**0.94**	0.89-0.99	

Combined Models						
All predictors (personal and socio-environmental)			0.63			0.44
Covariates only (cohort, race, weight status)			0.21			0.07

Note. *N *= 315 for boys, 764 for girls. All models are adjusted for cohort, race/ethnicity, and weight status from Time 1 to Time 2. *R*^2 ^= percent variance explained by separate and combined predictor models. Each of 12 single predictor models includes predictor, change in predictor, and covariates. Combined models include all personal, all socio-environmental, or all predictors as well as change in predictor and covariates. Statistically significant odds ratios (*p *< .05) are shown in **bold **type.

For socio-environmental factors, persisters reported relatively more peer dieting (boys' OR = 1.47, girls' OR = 1.56), weight teasing (boys' OR = 1.61, girls' OR = 1.35), and weight loss article reading (boys' OR = 1.96, girls' OR = 1.79); for girls only, greater parental weight concern also was associated with persistence (OR = 1.43). As with the findings for UWCB initiation, Time 1 weight concerns emerged as the strongest predictor of UWCB persistence at Time 2.

In addition, changes in most personal and socio-environmental factors from Time 1 to Time 2 were associated with persistent UWCB for all adolescents, with odds ratios comparable to those for the individual predictors at Time 1. For girls only, decreased self-esteem (personal, OR = 0.84) and family connectedness (socio-environmental, OR = 0.94) and increased parental concerns, peer dieting, and weight loss article reading (all socio-environmental) from Time 1 to Time 2 were associated with persistence of UWCB. As with the individual Time 1 predictor values, increases in weight concerns from Time 1 to Time 2 had the strongest association with UWCB persistence (boys' OR = 4.55; girls' OR = 3.03).

Examination of *R*^2 ^values in Table 2 indicates that, as with UWCB initiation, the block of personal factors had greater explanatory value with regard to UWCB persistence for both boys and girls, relative to the socio-environmental factors (*R*^2 ^= 0.53 and 0.41 for personal factors and *R*^2 ^= 0.33 and 0.19 for socio-environmental factors for boys and girls, respectively). Again, the largest contributor to UWCB persistence from individual predictor models was the weight concerns model (*R*^2 ^= 0.42 for boys, 0.26 for girls). In each individual model, the remaining personal predictors had only one-quarter to one-half the explanatory strength in explaining UWCB persistence. Among the individual socio-environmental factors, peer dieting, weight teasing, and weight loss article reading were comparably strong predictors of UWCB persistence for boys (*R*^2 ^= 0.25, 0.25, and 0.27, respectively). For girls, none of the socio-environmental predictors reached a predictive value above *R*^2 ^= 0.12. A model with all predictors entered (including all personal and socio-environmental predictors and covariates) had the strongest explanatory value (*R*^2 ^= 0.63 for boys and 0.44 for girls; see Combined Models section at bottom of Table 2).

## Discussion

The present study examined predictors of initiation or persistence of UWCB over a five-year period among adolescent males and females. The main intent of this study was to extend previous work on predictors of UWCB [9,24] by considering initiators and persisters separately (as compared to never-engagers or those who ceased UWCB, respectively), and by examining these links longitudinally. Our intent in doing so was to elucidate mechanisms of UWCB status to answer questions about how to build a program to prevent UWCB in adolescence; in particular, we were most interested in knowing whether prevention efforts to address UWCB among adolescents at different stages of the behaviour continuum during this transition period (i.e., those who never engage or those who quit, vs. those who start later in adolescence or those who persist throughout adolescence) might share common ground, thus streamlining intervention efforts in the field. Results suggest that a common set of personal and socio-environmental concerns underlie initiation and persistence of UWCB for girls and boys during adolescence

The rates of persistence of UWCB (by the end of the study, 44.4% of boys and 75.8% of girls had ever engaged in these behaviors) highlight the pressing need for UWCB prevention, as these high percentages suggest that the behaviours may be difficult to stop once engagement has begun. Our findings suggest that prevention of UWCB within a single program aimed at all adolescents may be suitable, thus allowing for development and dissemination of prevention programs in an efficient, broadly reaching manner.

Data in the present study suggest that similar patterns of personal and socio-environmental concerns, and changes in these factors over time, were associated significantly with initiation and persistence of UWCB for boys and girls. These results strengthen previous findings in this area with regard to the contributions of weight concerns of adolescents [17,18,24] and parents [21], peer influence [14,15,20,23], self-esteem [14,15,22], and weight loss magazine exposure [20] in the development and maintenance of UWCB in adolescence. Examination of the variance explained by each of these factors indicates that adolescents' concerns about weight emerged as the strongest predictor of engagement in UWCB in all models, suggesting that weight concern is a prime target for development of intervention efforts. In sum, the results presented here point to key contributors that should shape the focus of future interventions for adolescent girls and boys, regardless of UWCB status.

Consideration of the predictive value of the variables indicates the importance of personal factors in explaining UWCB initiation and persistence for both boys and girls. Results are consistent with our earlier conceptualization of personal factors, particularly weight concerns, as more proximal contributors to the predictive pathway for UWCB [24]; these findings further our previous work by suggesting that pathways are consistent regardless of UWCB status (initiation or persistence) and should be key components of UWCB prevention program development.

Gender patterns were comparable, in that personal factors were stronger contributors than socio-environmental factors for both initiation and persistence of UWCB for either boys or girls. However, some differences did emerge. Whereas the pattern of percent variance explained by classes of variables (personal > socio-environmental) was consistent across gender, personal factors were relatively stronger predictors of initiation for girls and relatively stronger predictors of persistence for boys. In addition, whereas socio-environmental factors were comparable predictors of UWCB initiation for boys and girls, these factors were relatively stronger predictors of UWCB persistence for boys. The patterns suggest that, even though the individual factors that contribute to UWCB are largely the same by gender (as evidenced by largely comparable odds ratios for individual predictors and change variables for boys vs. girls), different processes may be involved along the pathways of initiation versus persistence of UWCB that depend on other gender-specific influences. Despite higher base rates for eating disorders among girls relative to boys [4,8], future studies should be sure to enrol adequate numbers of boys and girls to examine this further.

Previous research has demonstrated that successful primary prevention programs designed to target unhealthy eating behaviours among adolescents have been built around components of self-esteem, body satisfaction, and healthy attitudes toward eating [41,42]. Findings from the current study provide additional data to support this style of intervention for secondary as well as primary prevention, as these components are comparably significant contributors not only to initiation of UWCB, but to persistence as well. Indeed, there has been some movement away from framing an intervention argument in terms of primary versus secondary prevention; rather, a trend toward a conceptualization of programs that are either universal (delivered to an entire community, such as a neighborhood or school) or targeted to high-risk populations has been advocated [43].

We argue here, based on our findings, that the problem of UWCB should be addressed universally (i.e., at schools), and that addressing it as such will simultaneously reach an appropriate high-risk population (i.e., adolescents). The patterns of association within the present data, taken with results from other studies by this research group and others [17,24,41,42] suggest that building such a program around the topic of weight concerns, the most salient contributor to UWCB initiation as well as maintenance, has the potential to maximize program benefits for all adolescents. By focusing on this sole prominent psychosocial construct, rather than on additional, more weakly associated concerns, programs may become more efficient as well.

Study strengths include a large, ethnically and socio-economically diverse sample with adequate data to examine the contrasts with respect to UWCB engagement; the prospective design also allowed for examination of comparisons over a key five-year period of adolescent developmental transition. A wide range of theoretically and empirically selected personal and socio-environmental factors were assessed [24,27], providing a rich framework within which to consider the study questions. Sample diversity and inclusion of boys and girls improves generalizability to a broader adolescent population.

Several limitations should be considered. This sample was drawn from one state in the U.S., which reduces generalizability despite the sample size and relative diversity as compared to the overall population of that state. Attrition may have affected outcomes; however, Time 2 data were weighted so that the demographic characteristics were comparable with the Time 1 sample. Self-reported measures of height and weight were used, which may have compromised the accuracy of these data. Several of the predictors (i.e., weight/shape importance, peer dieting, weight teasing, weight loss article reading), were assessed using single items to reduce response burden; the brevity of these measures may have narrowed our ability to assess these constructs fully. The study assessed UWCB by self-report, rather than by collecting clinical eating disorder measures, and aggregated UWCB for examination. An earlier analysis demonstrated that boys and girls who reported any UWCB (the assessment approach applied here) were significantly more likely to engage in extreme examples of UWCB (including vomiting or use of laxatives or diet pills to control weight), more likely to report binge eating with loss of control, and more likely to report having been diagnosed with an eating disorder [8]. Given the severe consequences of eating disorders and their associations with the mere presence of self-reported UWCB, we chose to study use of UWCB versus more strict definitions of disordered eating to broaden the potential impact of our findings.

## Conclusion

Future studies are needed to replicate these findings across a wider range of the adolescent population, as well as to develop and test programs constructed around the significant findings observed here. Results emphasize the importance of continued focus on improving psychosocial health and the social environment of adolescents to reduce the risk of developing disordered eating, decrease excessive preoccupation with weight, and contribute to programs that enhance self-acceptance during the adolescent transition.

## Competing interests

The authors declare that they have no competing interests.

## Authors' contributions

JAL drafted the manuscript. MMW performed the statistical analyses and contributed to the Data Analysis and Results sections of the manuscript. JH contributed to the literature review and helped to edit the manuscript. DNS conceived of the study, secured grant funding for the project, and helped to edit the manuscript.
